# Skeletal muscle autophagy and mitophagy in endurance-trained runners before and after a high-fat meal

**DOI:** 10.1016/j.molmet.2017.10.006

**Published:** 2017-10-21

**Authors:** Michael D. Tarpey, Kevin P. Davy, Ryan P. McMillan, Suzanne M. Bowser, Tanya M. Halliday, Nabil E. Boutagy, Brenda M. Davy, Madlyn I. Frisard, Matthew W. Hulver

**Affiliations:** 1 Department of Human Nutrition, Foods, and Exercise, Virginia Tech, Blacksburg, VA, USA; 2 Metabolic Phenotyping Core, Virginia Tech, Blacksburg, VA, USA; 3 Fralin Translational Obesity Research Center, Virginia Tech, Blacksburg, VA, USA

**Keywords:** Metabolic flexibility, Autophagy, Mitophagy, Endurance training, Skeletal muscle

## Abstract

**Objective:**

We tested the hypothesis that skeletal muscle of endurance-trained male runners would exhibit elevated autophagy and mitophagy markers, which would be associated with greater metabolic flexibility following a high-fat meal (HFM).

**Methods:**

Muscle biopsies were collected to determine differences in autophagy and mitophagy protein markers and metabolic flexibility under fasting conditions and 4 h following a HFM between endurance-trained male runners (n = 10) and sedentary, non-obese controls (n = 9).

**Results:**

Maximal oxygen consumption (ml·kg·min^−1^) was approximately 50% higher (p < 0.05) in endurance-trained runners compared with sedentary controls (65.8 ± 2.3 and 43.1 ± 3.4, respectively). Autophagy markers were similar between groups. Mitophagy and mitochondrial dynamics protein markers were significantly higher in skeletal muscle of endurance-trained runners compared with sedentary controls in the fasted state, although unaffected by the HFM. Skeletal muscle metabolic flexibility was similar between groups when fasted (p > 0.05), but increased in response to the HFM in endurance-trained athletes only (p < 0.005). Key mitophagy markers, phospho-Pink1^Thr257^ and phospho-Parkin^S65^ (r = 0.64, p < 0.005), and phospo-Parkin^Ser65^ and phospho-Drp1^Ser616^ (r = 0.70, p < 0.05) were correlated only within the endurance-trained group. Autophagy and mitophagy markers were not correlated with metabolic flexibility.

**Conclusion:**

In summary, mitophagy may be enhanced in endurance-trained runners based on elevated markers of mitophagy and mitochondrial dynamics. The HFM did not alter autophagy or mitophagy in either group. The absence of a relationship between mitophagy markers and metabolic flexibility suggests that mitophagy is not a key determinant of metabolic flexibility in a healthy population, but further investigation is warranted.

## Highlights

• Basal autophagy is similar in skeletal muscle of endurance-trained and sedentary males.
• Trained and sedentary skeletal muscle autophagy is unaltered following high-fat meal.
• Mitophagy activity is greater in endurance-trained than sedentary skeletal muscle.
• Greater mitophagy is related to increased metabolic flexibility after high-fat meal.
• Trained and sedentary skeletal muscle mitophagy is unaltered following high-fat meal.

## 1 Introduction

The maintenance of a healthy, functional mitochondrial network requires turnover through regulated shifts in the balance between fission and fusion and mitochondrial biogenesis and mitophagy [1]. Concerted action between mitophagy and autophagy, the more general degradation pathway, selectively isolates and eliminates damaged or dysfunctional mitochondria, maintaining overall network quality and function. Impaired skeletal muscle mitochondrial function is a hallmark of obesity, insulin resistance, and type II diabetes. Skeletal muscle from obese, insulin resistance, and type II diabetic individuals is characterized by impaired mitochondrial function, which includes fewer and smaller mitochondria [2], [3], [4], [5], reduced transport chain content [6], [7], [8] and gene expression [9], [10], and lower oxidative capacity [3], [11], [12], [13], [14]. This implies that mitochondrial quality control is either insufficient or defective in these disorders. The latter may have important implications for disease progression.

Metabolic inflexibility, defined as diminished capacity to adjust substrate oxidation in response to changes in substrate availability [15], [16], has been implicated in the pathogenesis of obesity and the development of insulin resistance. For example, the relative inability of skeletal muscle to coordinate compensatory increases in fat oxidation following lipid influx may lead to the accumulation of fat and lipid intermediates and, subsequently, a decline in insulin sensitivity [15], [17]. Sedentary behaviors are associated with reduced metabolic flexibility [18] and are considered a prominent factor in the etiology of obesity, insulin resistance, and type II diabetes [19], [20]. Conversely, exercise training is associated with improved metabolic flexibility and insulin sensitivity in obese [21], [22] and type II diabetic patients [23], [24].

The increases in metabolic flexibility observed with endurance training have been attributed to enhanced mitochondrial respiration in human skeletal muscle [25]. Similarly, restoration of skeletal muscle mitochondrial function in type II diabetic patients is accompanied by increased metabolic flexibility [24]. Besides highlighting potential disparities in skeletal muscle mitochondrial quality between endurance-trained and sedentary individuals, as well as in those with metabolic disease, these findings also imply a causative link between mitochondrial function and metabolic flexibility. While the direction of causation remains controversial, restoration of metabolic flexibility in type II diabetic patients was associated with increased mitochondrial content [24]. Accordingly, metabolic inflexibility in obesity-associated insulin resistant individuals was correlated to reduced intermyofibrillar mitochondrial content, which could not be accounted for by differences in mitochondrion size, muscle fiber distribution, or maximal aerobic capacity [26]. Together, these findings indicate that while mitochondrial content is a contributor to metabolic flexibility, in of itself, it may not be the major determinant. Instead, factors governing the mitochondrial population and quality may provide an alternative explanation. While the relationship between endurance exercise and mitochondrial biogenesis in skeletal muscle has been widely studied [27], [28], [29], [30], the role of autophagy and mitophagy is not well understood.

Chronic endurance exercise training leads to increased markers of basal autophagy and mitophagy in murine skeletal muscle [29], [31]. Meanwhile, single bouts of endurance exercise stimulate mitophagy in a fed state-dependent manner in endurance-trained human skeletal muscle in the absence of autophagy activation [32]. When the same endurance exercise bout was completed following a fast, the onset of mitophagy activation was delayed [32]. Ultra-endurance exercise, when conducted in a fed state, has been shown to increase markers of both autophagy and mitophagy activity in endurance-trained human skeletal muscle [33], [34]. It remains unclear whether autophagy and mitophagy regulation differs in skeletal muscle of endurance-trained compared to sedentary individuals. Diet also modulates skeletal muscle autophagy. High-fat diets have been associated with lipid-induced insulin resistance and reduced basal autophagy activity in murine skeletal muscle [1]. If, and how high-fat feeding modulates autophagy and mitophagy in human skeletal muscle is unknown.

The focus of the current study was to investigate whether endurance-trained runners exhibit elevated markers of autophagy and mitophagy in skeletal muscle compared to non-obese, sedentary controls and if the groups adjust autophagy and mitophagy regulation similarly following a high-fat meal (HFM). Finally, we sought to establish whether markers of autophagy and mitophagy in the fasted state and following a high-fat meal were related to skeletal muscle metabolic flexibility and oxidative capacity.

## 2 Materials and methods

### 2.1 Participants

Nine healthy, non-obese, sedentary (<2 days, 20 min/day of low-intensity physical activity) males and 10 endurance-trained (≥5-h running per week, and 2 competitions in the past 12 months) male runners aged 18–45 years completed the study. Participants were weight stable (<±2.5 kg) for the past 6 months with a BMI > 18 or < 30 kg/m^2^ and were not taking any medications or supplements known to affect study measures. All participants had blood pressure <140/90 mmHg, fasting glucose <126 mg/dL, total cholesterol <240 mg/dL or triglycerides <300 mg/dL, and percentage of habitual calorie intake composed of <40% fat and <15% saturated fat. Participants were non-smokers with no personal history of metabolic or cardiovascular disease. All study procedures were approved by the Virginia Tech Institutional Review Board. Prior to participation, all procedures, benefits, and any potential risks associated with the study were explained to the participants before written consent was provided.

### 2.2 Experimental design

**Figure 1:**
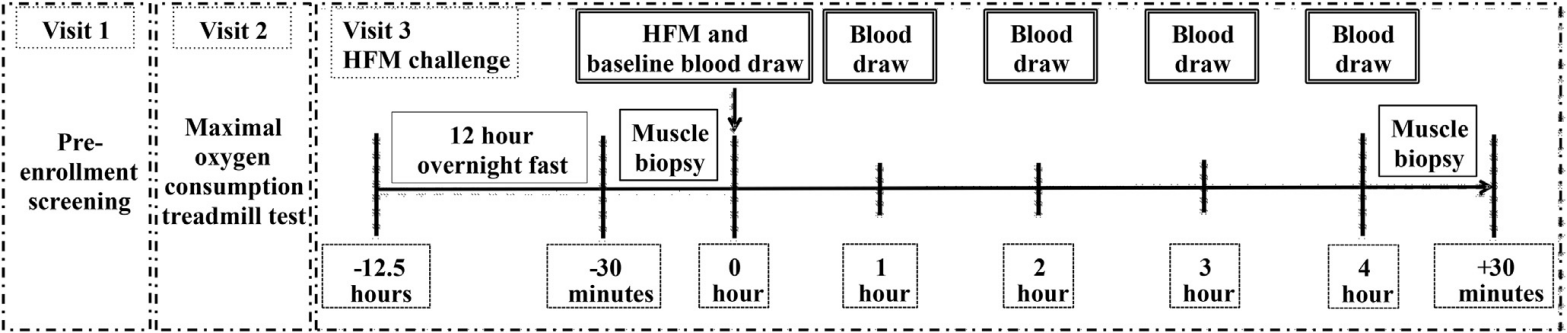
Schematic of study design. Participants completed a pre-enrollment screening prior to completing a maximal oxygen consumption test. Participants fasted for 12-h overnight prior to a baseline skeletal muscle biopsy taken at least 36-h after last exercise bout. Participants consumed a HFM and rested for 4-h before completing a second follow-up skeletal muscle biopsy. Blood was drawn every hour. HFM, high-fat meal.

Following successful completion of screening procedures, all participants underwent a maximal treadmill test to volitional exhaustion to determine maximal oxygen consumption (VO_2max_). Participants refrained from exercise for 36-h prior to a HFM challenge and muscle biopsies. Muscle biopsies were taken from the vastus lateralis following a 12-h overnight fast and 4-h after a HFM for assessment of markers of skeletal muscle autophagy, mitophagy, and metabolic flexibility. A schematic of the study design is presented in Figure 1.

### 2.3 HFM challenge

The HFM consisted of two sausage, egg, and cheese biscuits containing of 58 g fat (24 g saturated fat), 52 g carbohydrate, 24 g protein and a total of 820 kcal. Participants were required to consume the HFM within 10 min and remain seated and awake for the duration of the meal challenge. Following the initial biopsy and prior to the HFM, an intravenous catheter was placed in an antecubital vein for baseline and hourly blood sampling. The biopsies taken before and following the meal were from right and left legs, respectively.

### 2.4 Measurements

#### 2.4.1 Body mass and composition

Body weight was measured to the nearest ±0.1 kg on a digital scale (Model 5002, Scale-Tronix, White Plains, NY). Height was measured to the nearest ±0.1 cm using a stadiometer. Body composition (total fat and fat-free mass) was analyzed by dual-energy x-ray absorptiometry (General Electric, Lunar Digital Prodigy Advance, software version 8.10e Madison, WI).

#### 2.4.2 Dietary assessment

Participants completed four-day food diaries for the assessment of dietary intake, as previously described [34].

#### 2.4.3 Maximal oxygen consumption

Maximal oxygen consumption was measured during graded treadmill exercise to exhaustion using open-circuit spirometry (TrueMax 2400, ParvoMedics). Standard criteria for achievement of valid maximal oxygen consumption were met [35]

#### 2.4.4 Muscle biopsies

Biopsies were taken from the vastus lateralis muscle as previously described [36]. Muscle used to assess metabolic flexibility was immediately placed in SET buffer (0.25 M Sucrose, 1 mM EDTA, 0.01 M Tris-HCl and 2 mM ATP) and stored on ice until homogenization (∼25 min). Muscle tissue used for western blotting was placed in ice-cold cell lysis buffer (50 mM Tris-HCl, EDTA 1 mM, NaCl 150 mM, SDS 0.1%, sodium deoxycholate 0.5%, igepel Ca 630 1%, pH 7.5) with halt protease and phosphatase inhibitor cocktail (Thermo Scientific, Pittsburg, PA), and 0.1 mM bafilomycin A (Invivogen, San Diego, CA), then snap-frozen in liquid nitrogen. Protein samples were stored at −80 °C for later analysis.

#### 2.4.5 Western blotting

Proteins (20-40ug) were separated on SDS-PAGE gels (Criterion TGX Stain-Free Gels, Bio-Rad, Hercules, CA) and transferred to PVDF membranes using a Trans-Blot Turbo Transfer System (Bio-Rad). PVDF membranes were blocked for 1-h at room temperature in 5% non-fat dry milk or 5% bovine serum albumin prior to overnight incubation at 4 °C with primary antibodies. Following primary antibody incubation, membranes were incubated for 1-h at room temperature with HRP-conjugated secondary antibodies. Proteins were visualized via chemiluminescence (Clarity Western ECL Substrate, Bio-Rad, or SuperSignal West Femto, Thermo Scientific), quantified using Image Lab Software (v5.2.1, BioRad) and normalized to total lane protein content. SED and ET samples were divided equally between blots and samples from different blots were prepared and processed in parallel, and normalized to control samples. Molecular weight was determined by Precision Plus Protein Unstained Standards (Bio-Rad).

Primary antibodies used were FoxO3a (cat# 17026), Mfn1 (cat# 57602), Mfn2 (cat# 56889), Parkin (cat# 15954), Pink1 (cat# 23707) (Abcam, Cambridge, MA), Beclin-1 (cat# 3738), Bcl-2 (cat# 2870s), LC3B (cat# 2775), ULK1 (cat# 4773) (Cell Signaling, Danvers, MA), total OXPHOS cocktail (cat# MS601) (MitoSciences, Eugene, OR), Drp1 (cat# 110-55288) (Novus Biologicals, Littleton, CO), SQSMT1/p62 (cat# 28359) (Santa Cruz Biotechnology, Dallas, TX) and phospho-specific primary antibodies against FoxO3a (Thr^32^, cat# 26649), Parkin (Ser^65^, cat# 154995) (Abcam, Cambridge, MA), Drp1 (Ser^616^, cat# 3455), ULK1 (Ser^555^, cat# 5869 – equivalent to human Ser^556^) (Cell Signaling, Danvers, MA), and Pink1 (Thr^257^, cat# 68-0057-100) (Ubiquigent, Dundee, Scotland, UK).

#### 2.4.6 Metabolic flexibility

Metabolic flexibility was assessed by measuring [1–14C] pyruvate oxidation (0.35 uCI·mL^−1^ – 1 mM cold) ± non-labeled BSA (0.5%) bound-palmitate (100 uM). Flexibility is denoted by the percent decrease in pyruvate oxidation in the presence of free fatty acid (e.g. a greater percentage is indicative of greater metabolic flexibility). Flexibility is expressed as the ratio of CO_2_ production with labeled pyruvate over CO_2_ production with labeled pyruvate in the presence of palmitate. Each skeletal muscle sample was minced ∼200 times with scissors, transferred to a glass homogenization tube and homogenized on ice using a Teflon pestle (12 passes at 150 RPM). The sample rested on ice for ∼30 s and the homogenization steps were repeated. The homogenate was transferred to an Eppendorf tube and fresh sample was used to measure pyruvate oxidation. Briefly, 80 uL of a 20-fold (wt:vol) diluted muscle homogenate was incubated with 320 uL of reaction media (pH 7.4). Final concentrations of the reaction media were in mmol per liter: sucrose, 100; Tris-HCl, 10; potassium phosphate, 5; potassium chloride, 80; magnesium chloride, 1; l-carnitine, 2; malate, 0.1; ATP, 2; coenzyme A, 0.05; dithiothreitol, 1; EDTA, 0.2; and bovine serum albumin, 0.3%. After 1hr of incubation at 37 °C, 200 μl of 45% perchloric acid were injected to stop the reaction and evolve ^14^CO_2_ from the reaction media. ^14^CO_2_ produced during the incubation was trapped in 400 uL of 1 M sodium hydroxide. Trapped ^14^CO_2_ was determined by liquid scintillation counting by use of 5 ml EcoLite liquid scintillation cocktail (MP Biomedicals, Santa Ana, CA) on the LS 6500 scintillation counter (Beckman Coulter, Pasadena, CA). Homogenate protein concentrations were determined spectrophotometrically using a bicinchoninic acid assay (Thermo Scientific).

#### 2.4.7 Enzyme activity assays

The activities of citrate synthase (CS) and β-hydroxyacyl-CoA dehydrogenase (BHAD) were determined from the reduction of DTNB over time and the oxidation of NADH to NAD^+^, respectively as previously described [37], [38].

#### 2.4.8 Plasma glucose analysis

Blood samples were collected in K_3_ EDTA BD vacutainers and immediately centrifuged at 4 °C for 15 min at 2500G. Plasma was stored at −80 °C. Plasma glucose concentrations were determined using a YSI Stat Plus glucose analyzer (model 2300, Yellow Springs Instruments, Yellow Springs, OH).

#### 2.4.9 Statistical analysis

Two-way repeated measures analysis of variance was used to determine differences between groups, time, and group × time interactions for protein and metabolic outcome measures. Multiple comparisons were performed using a Tukey post-hoc analysis. Additionally, metabolic flexibility data were further analyzed using paired-samples t-tests. Independent t-tests were used to compare group characteristics and percent change in protein levels from pre- and post-meal time points between groups. Pearsons Product Moment correlations were used to assess associations between variables of interest. Data that did not follow a normal distribution were log base 10 or square root transformed as required to normalize data. Protein content data were available for 9 ET participants and 9 SED participants. All data are expressed as means ± standard error of the mean (SEM). The significance level was set *a priori* at α = .05.

## 3 Results

### 3.1 Participant characteristics

Participant characteristics are shown in Table 1. Age, body mass, BMI, and fasting glucose concentrations were similar between sedentary and endurance-trained groups. Body fat percentage (p = 0.001) and fasting serum triglyceride concentrations (p = 0.035) were lower in endurance-trained compared with sedentary participants. VO_2max_ was significantly higher in endurance-trained compared with sedentary individuals. Energy (kcal), fat (g) and carbohydrate (g) intake was greater for the endurance-trained than sedentary individuals (p = 0.031, 0.005 and 0.013, respectively); however, the proportion of energy intake as dietary fat (p = 0.117) and carbohydrate (p = 0.325) was similar between groups.

**Table 1:** Participant characteristics.

Characteristics	Sedentary	Endurance
Age (yrs)	23.2 ± 1.3	26.5 ± 2.5
Body mass (kg)	76.4 ± 4.7	71.0 ± 1.7
BMI	24.6 ± 1.0	22.5 ± 0.8
Body fat percentage	26.3 ± 3.0	14.4 ± 1.3^###^
VO_2max_ (ml·kg·min^−1^)	43.1 ± 3.4	65.8 ± 2.3^###^
Fasting glucose (mg·dL^−1^)	86.9 ± 2.2	79.1 ± 4.8
Fasting triglycerides (mg·dL^−1^)	103.1 ± 13.7	68.3 ± 7.4^#^
**Mean daily dietary intake**		
Energy intake (kcal)	2346 ± 207	3012 ± 194^#^
Fat intake (g)	90.0 ± 8.8	122.7 ± 5.2^##^
Fat intake (% of kcal)	34.1 ± 1.3	37.3 ± 1.4
Saturated fat intake (g)	28.9 ± 3.3	36.6 ± 2.8
Saturated fat intake (% of kcal)	10.7 ± 0.4	11.2 ± 0.9
CHO intake (g)	240.3 ± 16.2	340.7 ± 36.7^#^
CHO intake (% of kcal)	41.8 ± 2.3	44.4 ± 1.9
Protein intake (g)	106.7 ± 10.4	122.1 ± 9.1
Protein intake (% of kcal)	18.4 ± 1.5	16.3 ± 0.8

Values are mean ± SEM. Significant training effect: #, p < 0.05; ##, p < 0.01; ###, p < 0.001.

### 3.2 Autophagy markers

**Figure 2:**
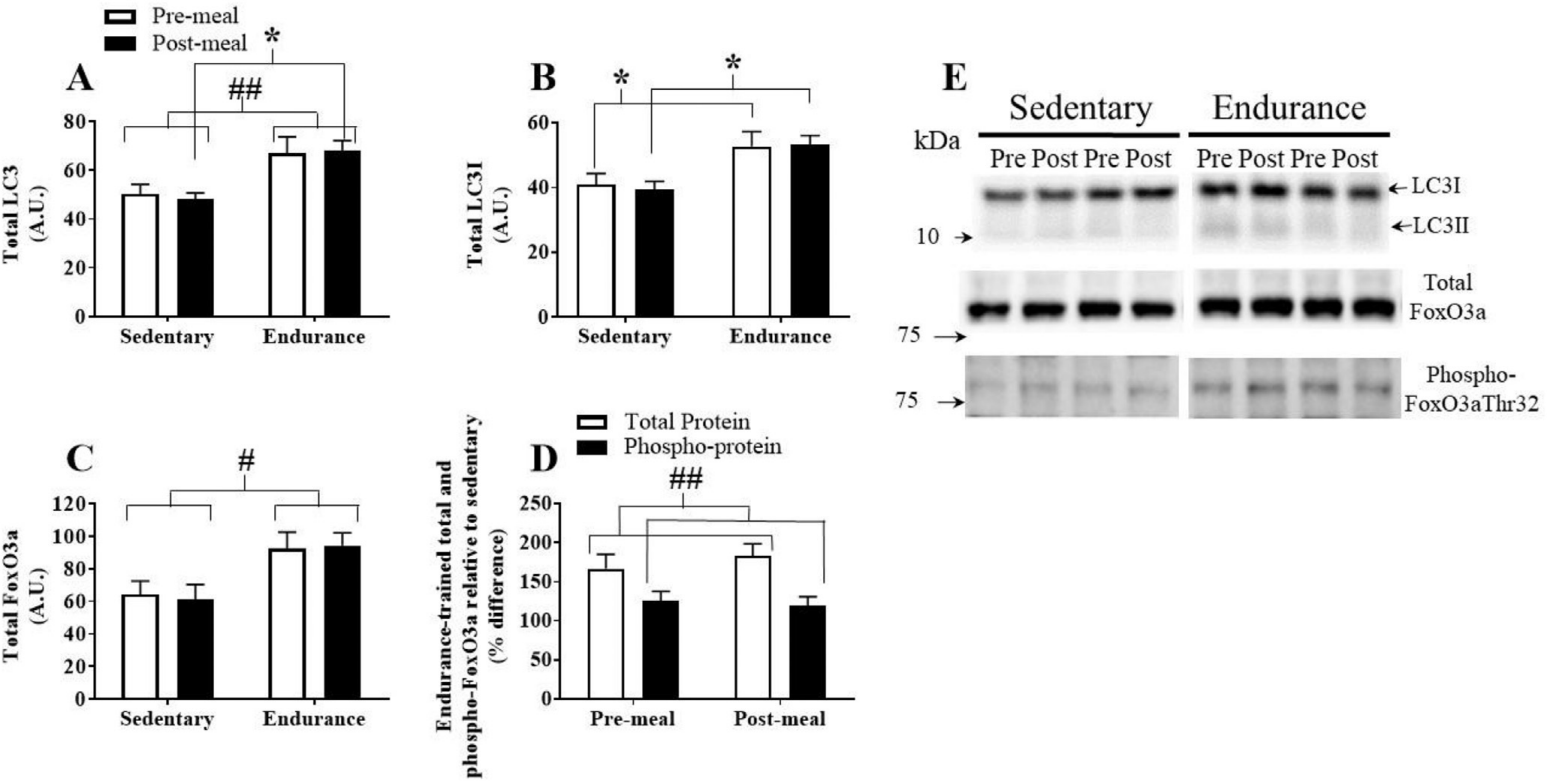
Assessment of markers of skeletal muscle autophagy activity. (A) Total LC3 protein content, (B) total LC3I protein content, (C) total FoxO3a protein content, (D) percent difference in total and phospho-FoxO3a^Thr32^ in ET participants relative to SED participants. (E) Representative western blots. Significant main effect of training: #, p < 0.05; ##, p < 0.01. Significant post-hoc training effect *, p < 0.05. Data are presented as mean ± SEM.

Total LC3 content tended to be higher in the fasting state (p = 0.081) and was significantly greater post-meal in endurance-trained compared to sedentary individuals (Figure 2A). There was no meal effect on total LC3. Endurance-trained skeletal muscle contained significantly greater LC3I content under fasting and fed conditions compared to sedentary skeletal muscle. (Figure 2B). LC3II protein content was not significantly different between groups (data not shown). There were no meal effects on total LC3, LC3I, or LC3II. SQSMT1/p62, a marker of autolysosome digestion [39], was similar between groups (data not shown). Anti-autophagy protein, Bcl-2, and pro-autophagy protein Beclin-1, were not significantly different between groups pre- and post-HFM (data not shown).

Skeletal muscle total ULK1 content, a modulator of skeletal muscle autophagy in humans [40], was not different between groups. There were no significant meal effects on total ULK1, although total ULK1 tended to be reduced following the HFM, independent of training status (p = 0.089) (data not shown). Autophagy-inducing phospho-ULK1^Ser556^ [40], [41] was similar between groups (data not shown) and also tended to be lower following the HFM, independent of training status (p = 0.072).

Skeletal muscle total FoxO3a, a transcriptional regulator of several autophagy and mitophagy proteins [8], was greater in endurance-trained than sedentary participants (p = 0.02, Figure 2C). Phospho-FoxO3a^Thr32^, an inhibitor of FoxO3a transcriptional activity [42], was similar between groups (data not shown). However, the relative difference in FoxO3a and phospho-FoxO3a^Thr32^ between endurance-trained and sedentary participants indicated a lower level of FoxO3a phosphorylation in endurance-trained skeletal muscle. The difference was most pronounced following the HFM (p = 0.005, Figure 2D). There were no meal effects on FoxO3a.

### 3.3 Mitophagy markers

**Figure 3:**
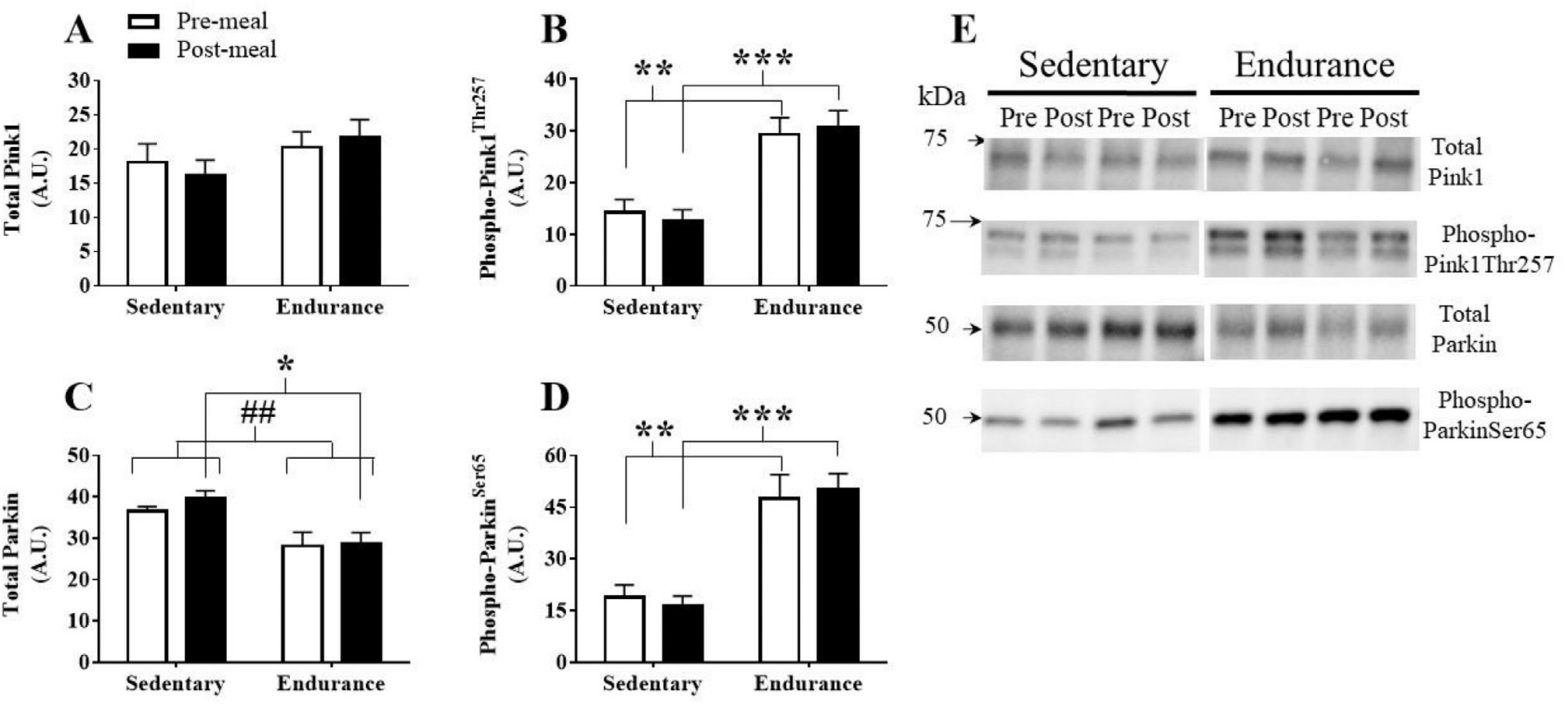
Assessment of skeletal muscle mitophagy marker protein content. (A) Total Pink1 protein content, (B) phospho-Pink1^Thr257^ protein content, (C) total Parkin protein content, (D) phospho-Parkin^Ser65^ protein content. (E) Representative western blots. Significant main effect of training: ##, p < 0.01. Significant post-hoc training effect: *, p < 0.05; **, p < 0.01; ***, p < 0.001. Data are presented as mean ± SEM.

Protein content of total Pink1, a mitophagy activator when phosphorylated at Thr257 [43], was similar between groups before and after the HFM (Figure 3A). Phospho-Pink1^Thr257^ content was greater in endurance-trained skeletal muscle in the fasted (p = 0.004) and fed state (p = 0.001) compared with sedentary participants, but was unaffected by the HFM (Figure 3B). The relative difference in total Pink1 and phospho-Pink1^Thr257^ protein content between groups confirmed a higher degree of Pink1 phosphorylation in endurance-trained skeletal muscle (p < 0.0001, data not shown).

There was a significant main training effect for greater total Parkin protein content in sedentary participants. The difference was pronounced following the HFM (p = 0.023, Figure 3C). Parkin promotes mitophagy following Pink1^Thr257^-dependent phosphorylation at Ser65 [43], [44]. Phospho-Parkin^Ser65^ was significantly greater in skeletal muscle of endurance-trained participants when fasted and following the HFM (Figure 3D). The relative difference in total Parkin and phospho-Parkin^Ser65^ between groups indicated a higher degree of Parkin phosphorylation in ET skeletal muscle (p < 0.0001, data not shown). Total and phosphorylated Parkin protein content were unaffected by the HFM.

### 3.4 Mitochondrial dynamics and ETC content

**Figure 4:**
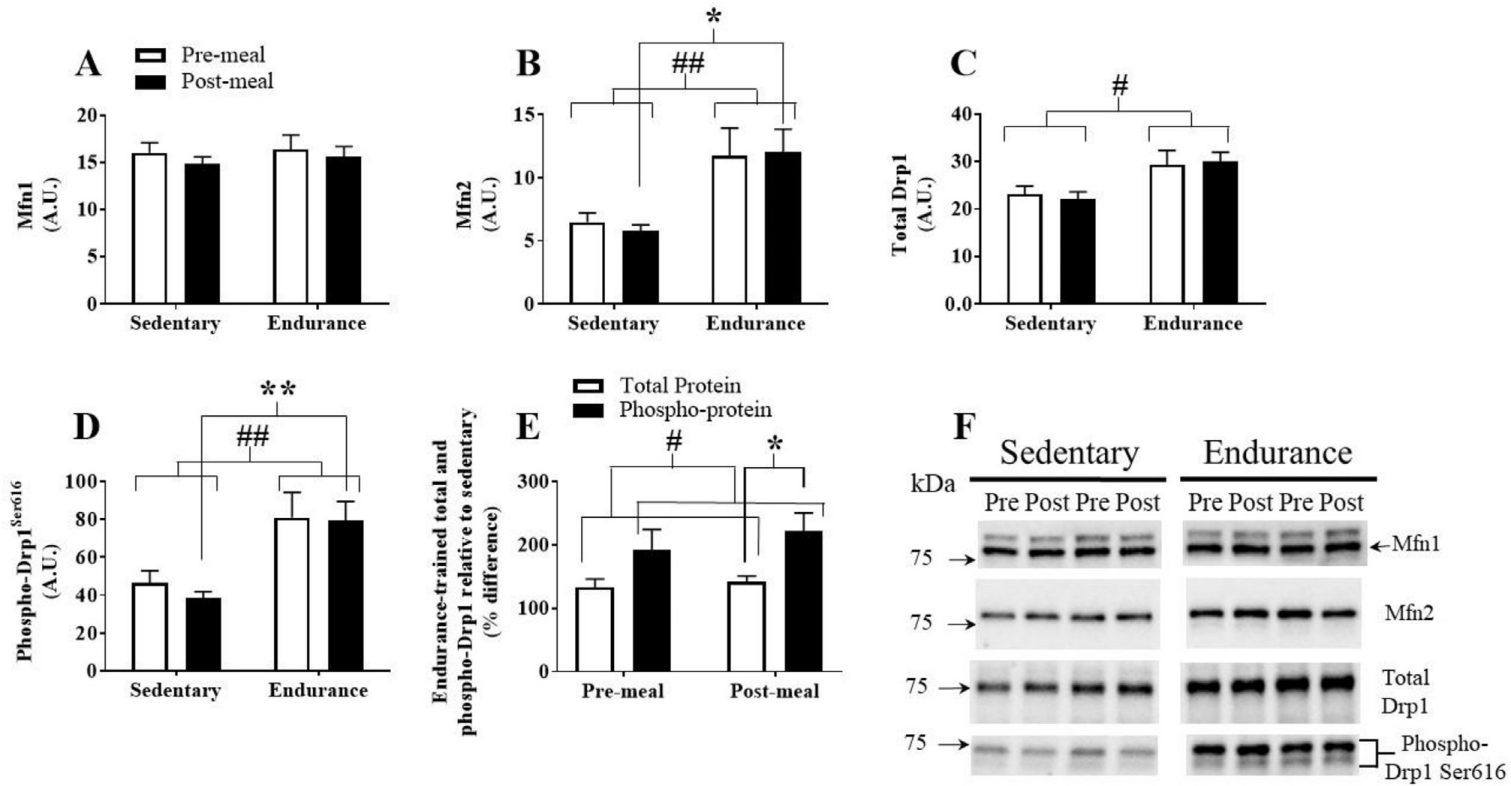
Assessment of skeletal muscle mitochondrial dynamic markers. (A) Total Drp1 protein content, (B) phospho-Drp1^Ser616^ protein content, (C) percent difference in total and phospho-Drp1^Ser616^ in ET participants relative to SED participants, (D) total Mfn1 protein content, (E) total Mfn2 protein content. (F) Representative western blots. Significant main effect of training: #, p < 0.05; ##, p < 0.01. Significant post-hoc training effect: *, p < 0.05; **, p < 0.01. Data are presented as mean ± SEM.

Mitochondrial fusion protein, Mfn1, was similar between groups before and after the HFM (Figure 4A). Conversely, there was a main training effect on Mfn2 protein content, which was greater in the endurance-trained group (p = 0.005). Mfn2 protein content tended to be higher in the fasting state (p = 0.071) and significantly higher following the HFM in the endurance-trained participants (Figure 4B). Mitochondrial fission protein, Drp1, which is activated following phosphorylation at Ser616 [45], was more abundant in the endurance-trained group (p = 0.02, Figure 4C). There was a main training effect on phospho-Drp1^Ser616^ content, which was greater in the endurance-trained group (p = 0.003). Phospho-Drp1^Ser616^ trended higher in the fasting state (p = 0.06) and significantly higher following the HFM (p = 0.005, Figure 4D). The relative difference in total Drp1 and phospho-Drp1^Ser616^ between groups indicated a higher degree of Drp1 phosphorylation in endurance-trained skeletal muscle (p < 0.002) with the effect most pronounced following the HFM (p = 0.048). There were no meal effects on Mfn1, Mfn2, or Drp1.

**Figure 5:**
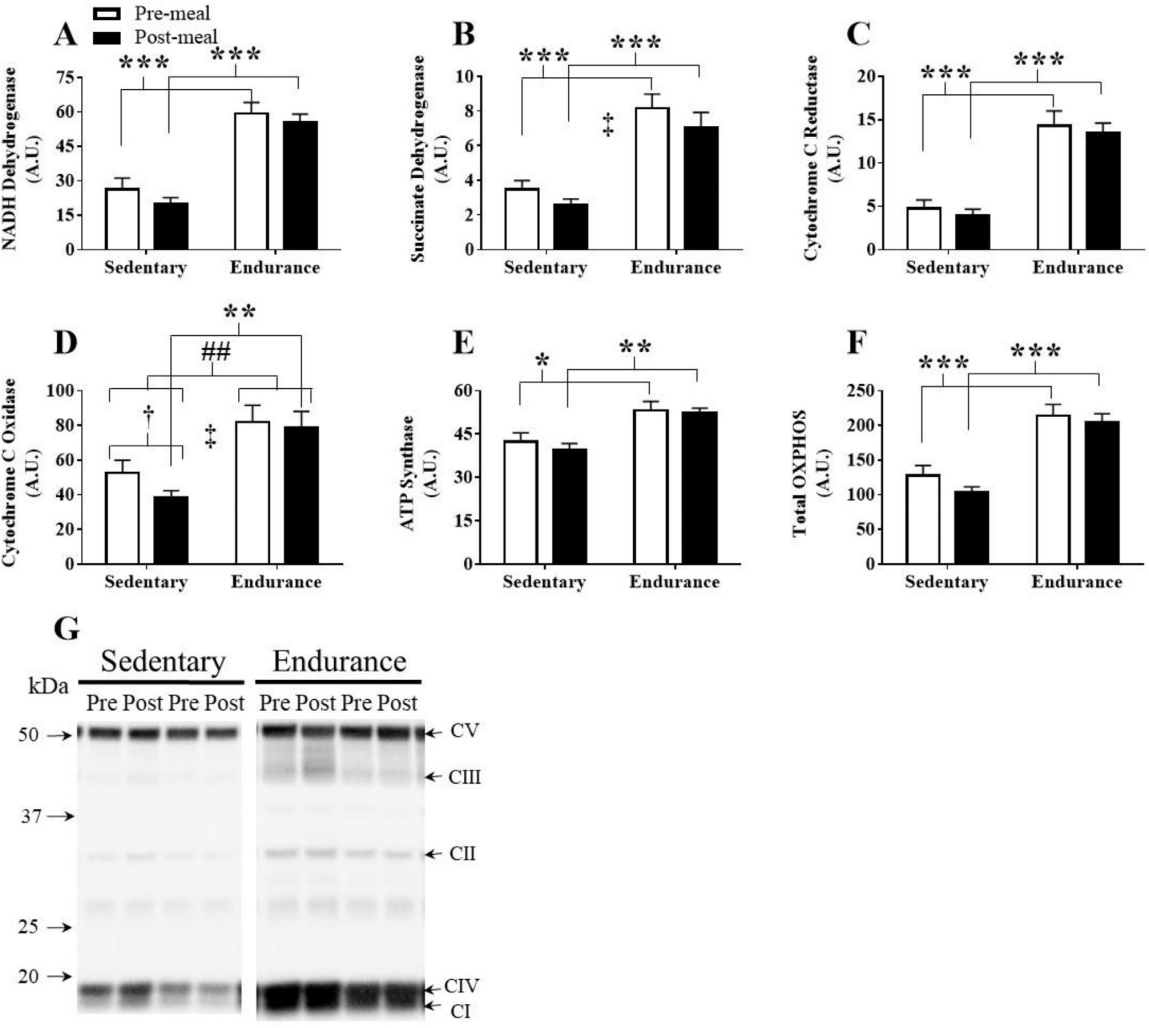
Assessment of skeletal muscle electron transport chain complexes. (A) NADH dehydrogenase (complex I) protein content, (B) succinate dehydrogenase (complex II) protein content, (C) cytochrome c reductase (complex III) protein content, (D) cytochrome c oxidase (complex IV) protein content, (E) ATP synthase (complex V) protein content, (F) total ETC protein content (sum of all ETC proteins). (G) Representative western blots. Significant main effect of training: ##, p < 0.01. Significant post-hoc training effect: *, p < 0.05; **, p < 0.01; ***, p < 0.001. Significant post-hoc within-group meal effect: †, p < 0.05. Meal effect, independent of training status; ‡, p < 0.05. Data are presented as mean ± SEM.

The ETC complexes I-V (Figure 5A–E, respectively) and total ETC protein content (sum of complexes I-V) (Figure 5F) were greater in skeletal muscle of endurance-trained participants. Complexes I, II, III, and V, and total ETC content were significantly greater in the fasting state and following the HFM. Complex IV protein content showed a main training effect, tending to be higher in endurance-trained compared to sedentary individuals during the fasting state (p = 0.069), and significantly higher following the HFM. Meal effects were detected following reductions in complex II (p = 0.040) and IV (p = 0.030), independent of training status. In response to the HFM complex IV was reduced within the sedentary group (p = 0.045).

### 3.5 Metabolic markers

**Figure 6:**
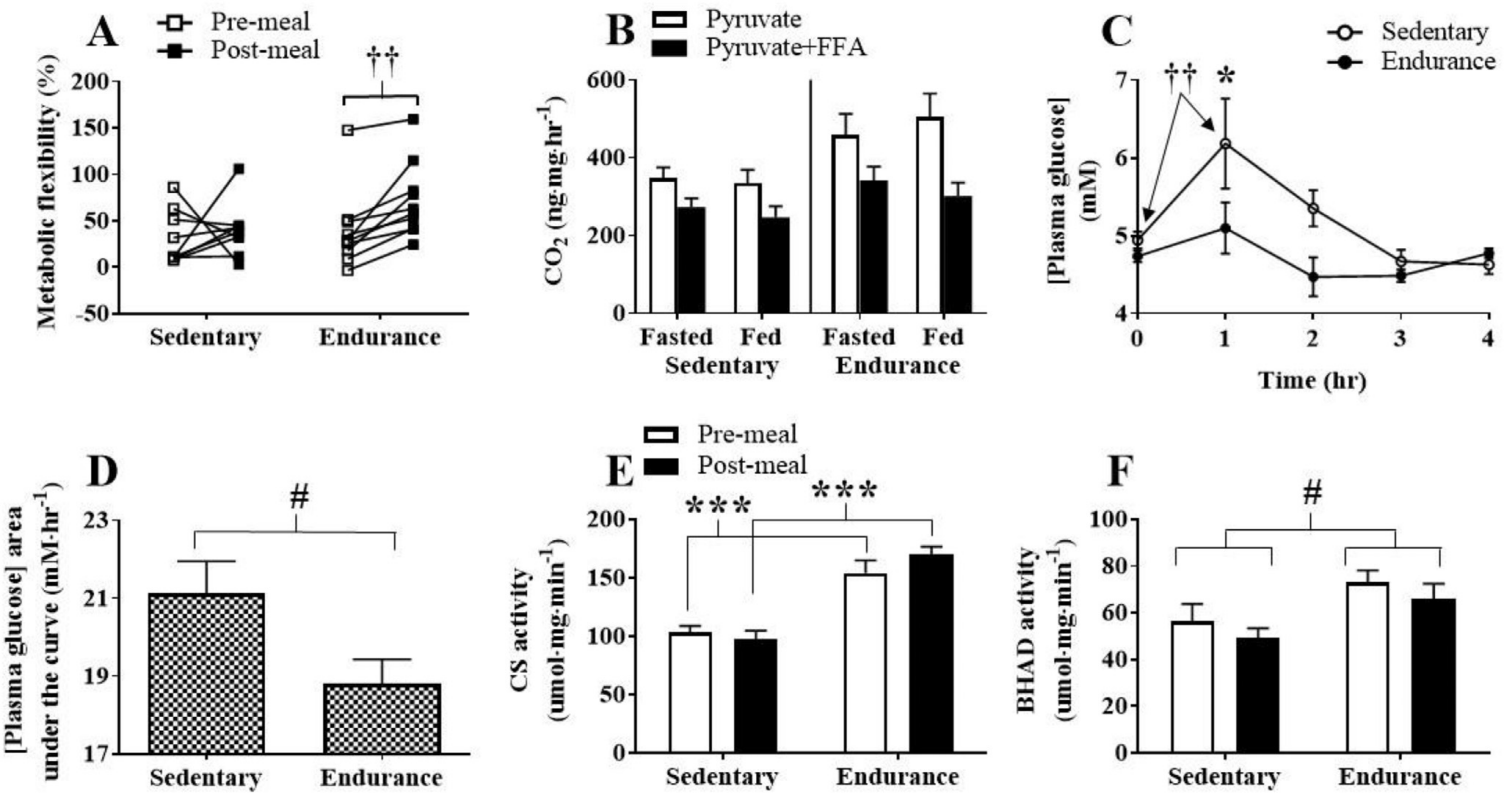
Assessment of markers of skeletal muscle metabolic function. (A) Individual participant metabolic flexibility; percent change in pyruvate oxidation in the presence of FFA, (B) muscle homogenate CO_2_ production in the presence of pyruvate, and pyruvate plus FFA (C) plasma glucose concentration in response to a high-fat meal, (D) plasma glucose area under the curve, (E) citrate synthase (CS) activity, (F) β-hydroxyacyl-CoA dehydrogenase (BHAD) activity. Significant main effect of training: #, p < 0.05. Significant post-hoc training effect: *, p < 0.05; ***, p < 0.001. Significant post-hoc within-group meal effect: ††, p < 0.01. Data are presented as mean ± SEM.

Skeletal muscle metabolic flexibility was not significantly different between groups in the fasted or fed state. However, metabolic flexibility increased following the HFM within the endurance-trained group (p = 0.002, Figure 6A). Accompanying CO_2_ production values for the metabolic flexibility assay are shown in Figure 6B. Plasma glucose was similar in the two groups in the fasting state, but significantly different 1 h post-HFM, and then similar throughout the remainder of the 4 h post-HFM period (Figure 6C). Sedentary participants significantly increased plasma glucose 1 h post-HFM, whereas plasma glucose of endurance-trained participants remained stable. Glucose area under the curve (AUC) during the 4 h post-HFM was significantly lower in the endurance-trained compared to sedentary group (Figure 6D). Citrate synthase activity was significantly greater in endurance-trained skeletal muscle before (p < 0.001, Figure 6E) and after the HFM (p < 0.001). There was a main effect for greater BHAD activity (p = 0.016, Figure 6F) in the endurance-trained compared to sedentary group. Citrate synthase and BHAD activity were not affected by the HFM in either group (Figure 6E and F).

### 3.6 Correlations

**Figure 7:**
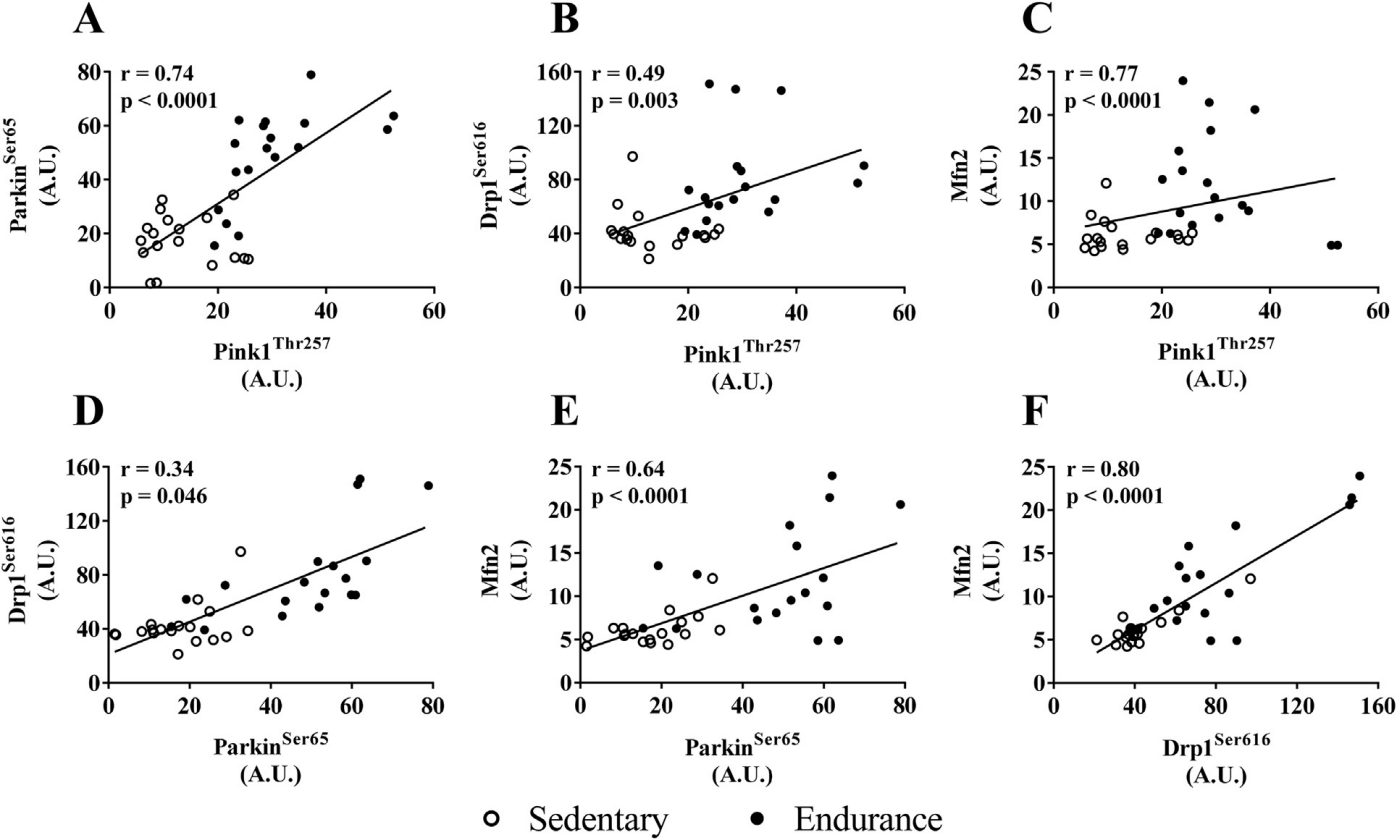
Correlations between skeletal muscle content of mitophagy and mitochondrial dynamic proteins. (A&B) phospho-Pink1^Thr257^ and phospho-Parkin^Ser65^, (C&D) phospho-Pink1^Thr257^ and phospho-Drp1^Ser616^, (E&F) phospho-Pink1^Thr257^ and Mfn2, (G&H) phospho-Drp1^Ser616^ and Mfn2. Correlations were based on fasted and post-HFM data points.

**Figure 8:**
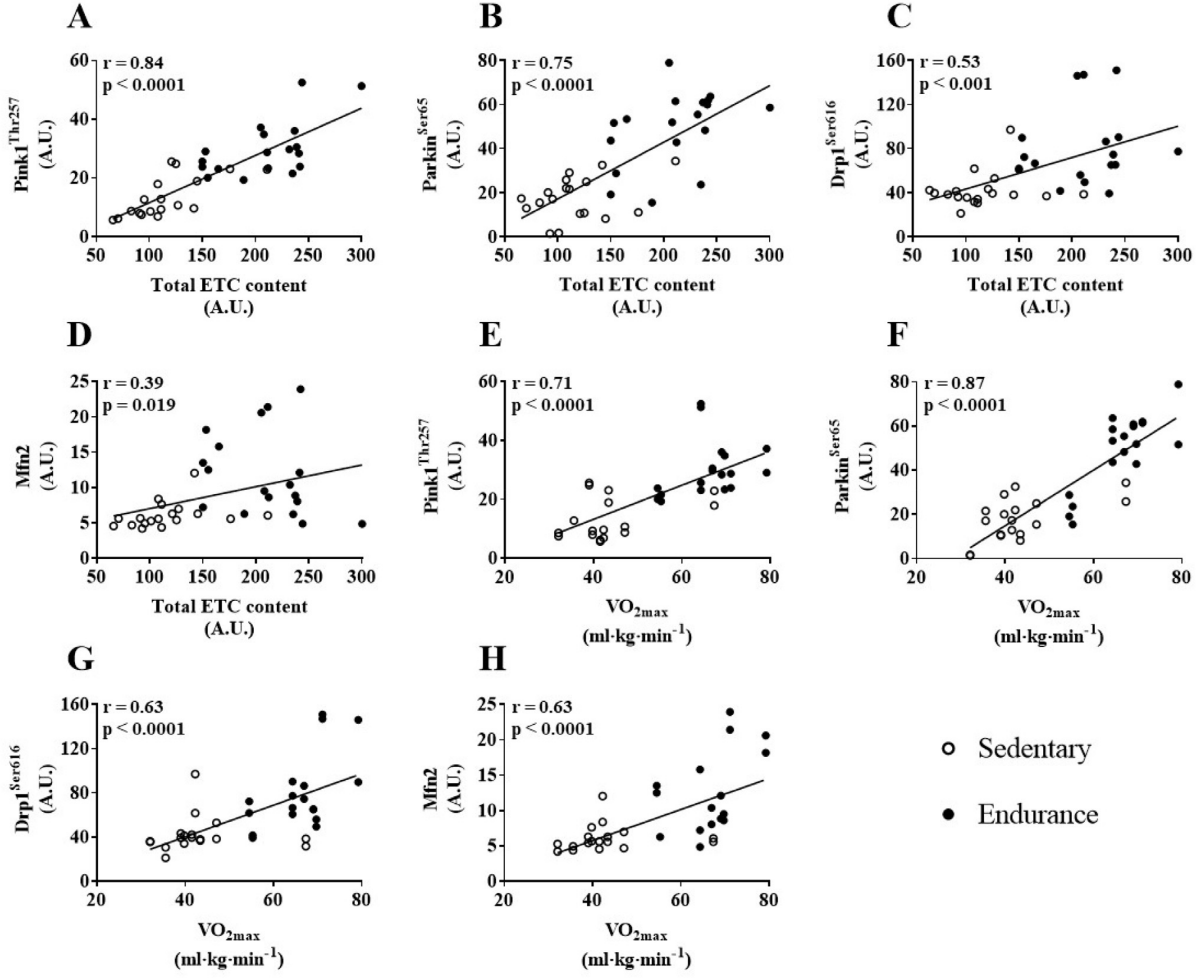
Correlations between skeletal muscle total ETC protein content and maximal oxygen consumption (VO_2max_) compared to content of mitophagy and mitochondrial dynamic proteins. (A&B) total ETC protein content and phospho-Pink1^Thr257^, (C&D) VO_2max_ and phospho-Parkin^Ser65^, (E&F) VO_2max_ and phospho-Drp1^Ser616^, (G&H) VO_2max_ and Mfn2. Correlations with protein data were based on fasted and post-HFM data points.

**Figure 9:**
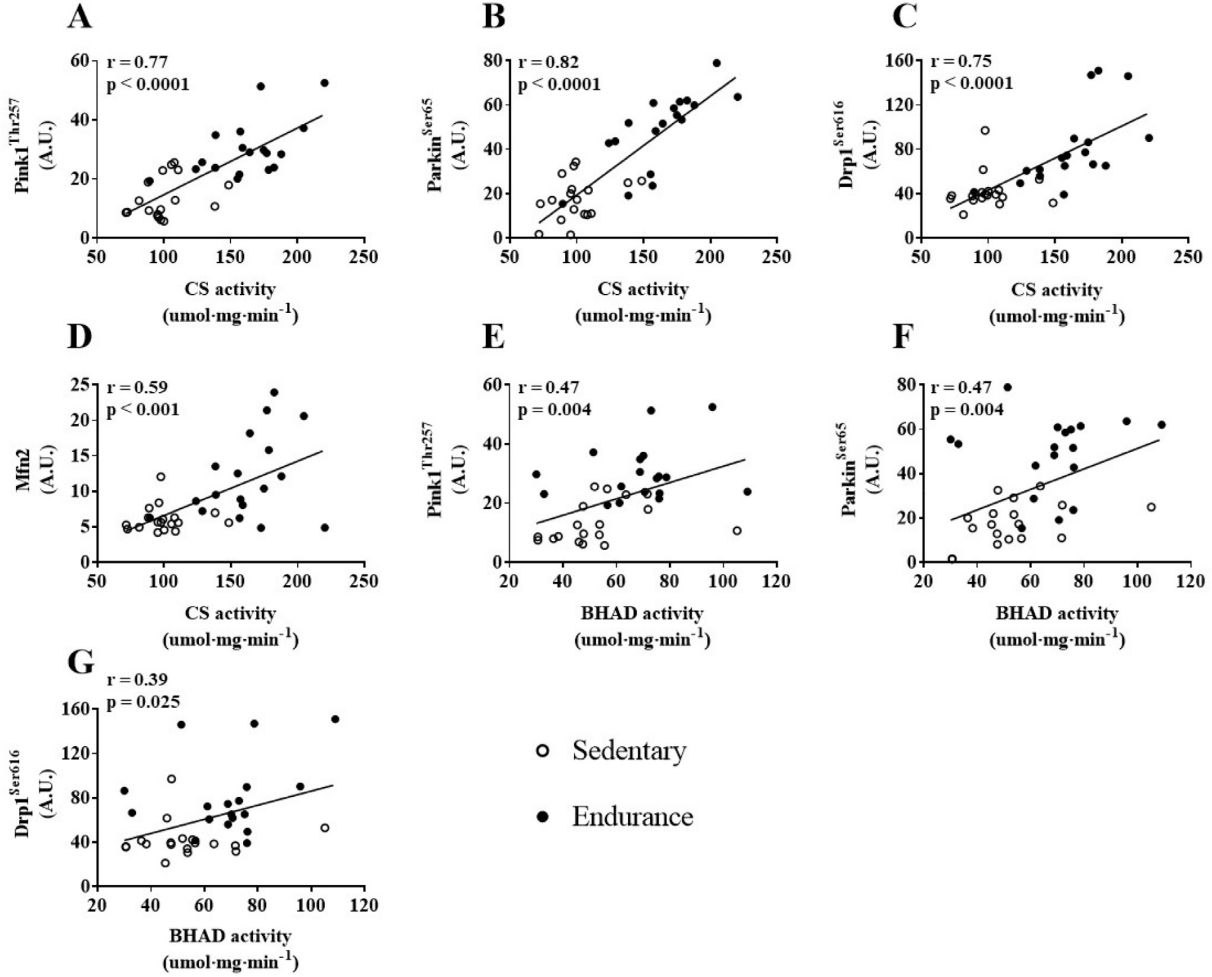
Correlations between skeletal muscle citrate synthase (CS) activity and β-hydroxyacyl-CoA dehydrogenase (BHAD) activity compared to content of skeletal muscle mitophagy and mitochondrial dynamic proteins. (A&B) CS activity and phospho-Pink1^Thr257^, (C&D) CS activity and phospho-Parkin^Ser65^, (E&F) CS activity and phospho-Drp1^Ser616^, (G&H) BHAD activity and phospho-Parkin^Ser65^. Correlations were based on fasted and post-HFM data point.

There were significant positive correlations between mitophagy and mitochondria dynamics markers, phospho-Pink1^Thr257^, phospho-Parkin^Ser65^, phospho-Drp1^Ser616^, and Mfn2 (Figure 7A–F). Total ETC protein content was correlated with VO_2max_ (r = 0.75 and p < 0.0001, data not shown), and both were significantly and positively correlated with phospho-Pink1^Thr257^, phospho-Parkin^Ser65^, phospho-Drp1^Ser616^, and Mfn2 (Figure 8A–H). There were no significant correlations observed between skeletal muscle metabolic flexibility and markers of autophagy or mitophagy in skeletal muscle. Citrate synthase activity was significantly and positively correlated with phospho-Pink1^Thr257^, phospho-Parkin^Ser65^, phospho-Drp1^Ser616^ and Mfn2 (Figure 9A–D, respectively), while BHAD was correlated with phospho-Pink1^Thr257^, phospho-Parkin^Ser65^, and phospho-Drp1^Ser616^ (Figure 9E–G).

## 4 Discussion

The primary finding of the current study was that skeletal muscle autophagy activity was not different between endurance-trained and sedentary participants in the fasted state or following a HFM. However, markers of mitophagy were higher in endurance-trained males in the fasted state and following the HFM compared with sedentary individuals. While skeletal muscle metabolic flexibility was not significantly different between sedentary and endurance-trained groups when fasted or following a HFM, there was a significant within-group increase in metabolic flexibility within the endurance-trained group in response to the HFM. Skeletal muscle mitophagy marker content was not associated with metabolic flexibility, suggesting the two may not be mechanistically linked.

Endurance training increases markers of basal autophagy in skeletal muscle of mice [31]. Acute endurance exercise bouts have been found to stimulate autophagy and mitophagy in skeletal muscle of trained [32], [33], [34] and untrained humans [40], but, until now, the impact of endurance-training remained unclear. Our present findings suggest that basal autophagy is not elevated in skeletal muscle of endurance-trained humans. While total LC3 and LC3I protein was greater in the endurance-trained group, it is unclear whether this translates to differences in autophagy regulation or activity because LC3 is degraded in the autolysosome [39]. Autophagy markers were unaltered in either group following a HFM, except for the relative phosphorylation status of FoxO3a, which was lower in endurance-trained skeletal muscle. Phosphorylation of FoxO3a at Thr32 retains FoxO3a in the cytoplasm, preventing its translocation to the nucleus and inhibiting the transcription of key autophagy/mitophagy proteins, including LC3 [42]. Endurance-trained skeletal muscle may therefore exhibit elevated transcription of particular autophagy and mitophagy proteins compared to sedentary individuals. Greater LC3I content in endurance-trained skeletal muscle may provide evidence of such a relationship. The correlative patterns between autophagy protein markers and participant characteristics were similar between groups, indicating that training status may not be a predictor of autophagy activity.

The current study is the first to utilize the phosphorylation status of Pink1 and Parkin as a method for the determination mitophagy activity in human skeletal muscle. Pink1 stabilizes and autophosphorylates at several residues in response to mitochondrial membrane depolarization, including Thr257 [43]. Phosphorylated Pink1^Thr257^ initiates E3 ligase activity of Parkin while simultaneously recruiting Parkin and ubiquitin to the outer mitochondrial membrane (OMM), a process facilitated by phosphorylation of Parkin and ubiquitin at Ser65 by phospho-Pink1^Thr257^ [43], [44]. The ubiquitin-associated domains provide docking sites for p62, thereby allowing the anchoring of mitophagy-tagged mitochondria to autophagosomes through the LC3-interacting region of p62 [46], [47]. The greater phospho-Pink1^Thr257^ and phospho-Parkin^Ser65^ content in the endurance-trained group suggests that skeletal muscle of endurance-trained individuals may exhibit heightened mitophagy activity. Phospho-Pink1^Thr257^ and phospho-Parkin^Ser65^ were also found to be positively correlated in endurance-trained, but not in sedentary skeletal muscle. Considering the mechanistic relationship between phospho-Pink1^Thr257^ and phospho-Parkin^Ser65^, this may be anticipated under conditions of greater mitophagy activation. Furthermore, Parkin is degraded in the lysosome during mitophagy [48]; thus, the lower total Parkin content in skeletal muscle of endurance-trained participants would also appear to support higher mitophagy activity.

Mitophagy pathways are closely aligned with those of mitochondrial dynamics. Mitochondrial fission, for example, appears to be a pre-requisite for mitophagy [49]. Downregulation of mitochondrial fission proteins Drp1 and Fis1 produces an elongated mitochondrial network that is resistant to mitophagy [49]. The greater Drp1 activity in endurance-trained skeletal muscle, based on a higher phospho-Drp1^Ser616^ content relative to total Drp1, may then reflect a pro-fission state that is primed for mitophagy. Therefore, it is interesting to note that phospho-Parkin^Ser65^ and phospho-Drp1^Ser616^ were positively correlated in endurance-trained, but not sedentary, skeletal muscle, further supporting an endurance training-induced upregulation of mitophagy.

Conversely, the elevated Mfn2 content of ET skeletal muscle could be interpreted as contradictory to the pro-mitophagy environment described thus far. Mfn2 is an important regulator of OMM fusion [50], which may provide a counterbalance to phospho-Drp1^Ser616^-induced mitochondrial fission and potentially an inhibitory influence on mitophagy. The fusion of mitochondria tagged for mitophagy is prevented by Parkin-mediated degradation of Mfn1 and Mfn2 [51], [52]. That Mfn1 content was similar between groups and Mfn2 is greater in endurance-trained skeletal muscle appears inconsistent with the higher phospho-Parkin^Ser65^ content in endurance-trained individuals. The role of Mfn2, however, may be more complex. In addition to mitochondrial fusion, Mfn2 aids in the ubiquitination and elimination of mitochondria by providing a receptor for phospho-Parkin^Ser65^ on the OMM [53]. Under such circumstances, the findings of the current study might provide additional evidence supporting greater mitophagy activity in endurance-trained skeletal muscle.

The mechanism(s) responsible for the elevated mitophagy signaling observed in endurance-trained skeletal muscle in the present study is unclear, but likely related to training-induced mitochondrial adaptations. An initiator of mitochondrial fission and Drp1 activity is the loss of mitochondrial membrane potential [54], the same mechanism responsible for the stabilization and autophosphorylation of Pink1 at Thr257 [43]. We did not assess mitochondrial membrane potential, although an inverse relationship between training status and mitochondrial membrane has previously been reported [55]. A chronic reduction in membrane potential may stabilize Pink1 in ET skeletal muscle, increasing mitophagy activity. Further investigation is required to support such a mechanism. The strong positive correlations between mitophagy markers and total ETC protein content and VO_2max_ as well as CS and BHAD activity support a relationship between mitophagy activity and oxidative capacity. Previous studies have indicated a similar relationship. Lira et al. [31], reported elevations in the mitophagy marker Bnip3 in the oxidative muscle fibers of mice compared to glycolytic fibers. SED mice overexpressing Pgc1-α exhibited the same elevation in Bnip3 [31] suggesting that increases in mitophagy markers may be a by-product of increased mitochondrial content and not the result of training per se. In the current study however, when normalized to citrate synthase activity and total ETC protein content, phospho-Pink1^Thr257^, phospho-Parkin^Ser65^, phospho-Drp1^Ser616^, and Mfn2 content remained significantly higher in endurance-trained skeletal muscle. Taken together, these observations suggest that differences between groups in mitophagy markers are not simply the result of greater mitochondrial content, but a specific adaptation to endurance training. It is important to note that the protein-based evidence for greater mitophagic activity in endurance-trained human skeletal muscle, while compelling, is nonetheless indirect since we cannot account for post-translational modification potentially that may regulate mitophagy pathways.

In addition to training status we also sought to investigate whether a HFM would affect mitophagy activity. High-fat meals are associated with increased ROS production and oxidative stress [56], [57]. ROS production and oxidative stress may modulate mitophagy by stimulating Pink1 and Parkin transcription [58], mitochondrial depolarization [59], and impairing Parkin function [60], [61], [62]. The lack of a HFM effect on the protein content of phospho-Pink1^Thr257^ and phospho-Parkin^Ser65^ in the skeletal muscle of either group suggest that a single HFM is not a modulator of mitophagy activity in endurance-trained or sedentary skeletal muscle.

A secondary objective of the study was to determine whether differences between groups in autophagy or mitophagy activity would be associated with altered metabolic flexibility. Metabolic flexibility has previously been positively correlated to mitochondrial content [24], [26]. Despite evidence of greater mitochondrial content in endurance-trained individuals based on CS activity and OXPHOS protein content, metabolic flexibility was not significantly different between groups in the fasted state or following the HFM. van de Weijer et al. [13] reported that in-vivo mitochondrial function, assessed via PCr recovery, was the major determinant of basal respiratory exchange ratio (RER) but was not a major contributor under insulin-stimulated conditions. If mitochondrial function was not compromised in the healthy, sedentary group then differences in metabolic flexibility may not be present, regardless of mitochondrial content.

However, endurance-trained participants significantly increased metabolic flexibility in response to the HFM, while sedentary participants did not. If elevated mitophagy activity in endurance-trained individuals assists in improving the overall quality of the mitochondrial reticulum it may not provide superior metabolic flexibility under low-stress, fasting conditions when compared to healthy, sedentary individuals but may enhance the metabolic flexibility of skeletal muscle following exposure to high-fat feeding. Previous reports of correlations between mitochondrial content and metabolic flexibility were conducted in type II diabetic patients and obesity-associated insulin resistant individuals and thus may not be applicable when studying healthy, sedentary populations if mitochondrial function and/or content is not compromised. The present study did not measure mitochondrial function and therefore cannot confirm if this was a factor. It may be worth noting though that complex IV (cytochrome oxidase) was significantly reduced in sedentary skeletal muscle in response to the HFM. Computer simulation models of mitochondrial function indicate that a deficiency in complex IV would compromise mitochondrial function, specifically ATP production [63]. This in turn may reduce the mitochondrial reticulum's capacity for reducing equivalents, triggering a range of negative feedback loops, which may ultimately limit the substrate switching potential of the muscle. Whether the disparate response in complex IV content between groups, following the HFM, is the result of differences in mitophagy activity/regulations is not clear.

In contrast, Dube et al. [25] reported significantly greater metabolic flexibility in endurance-trained humans compared with lean sedentary controls in response to a lipid infusion. The discrepant findings between our study and Dube et al. [25] are likely the result of different methodologies. The current study employed a HFM containing 58 g fat (24 g saturated fat) consumed within 10 min to induce lipid overload and assayed skeletal muscle homogenate to determine metabolic flexibility. In contrast, Dube et al. [25] used a 6hr exogenous lipid infusion protocol [1.5 ml/min, 20% fat emulsion (30% soybean oil, 1.2% egg yolk phospholipids, 1.7% glycerol, water)] and measured whole body substrate utilization via indirect calorimetry. Nonetheless, their previous report [25] is consistent with our finding that endurance-trained individuals possess an augmented capacity to regulate substrate oxidation in response to lipid stress. Dube et al. [25] also noted heightened mitochondrial respiration in endurance-trained skeletal muscle relative to sedentary individuals. The authors [25] concluded that greater metabolic flexibility and insulin sensitivity in endurance-trained individuals following an exogenous lipid infusion was related to superior mitochondrial performance. The current study did not measure mitochondrial respiration, although such measures may have provided greater clarity on the interaction between mitophagy and metabolic flexibility between sedentary and endurance-trained individuals. Taken together, these findings support the need for further studies investigating the relationship between mitophagy and mitochondrial respiratory function in skeletal muscle as a mechanism for sustaining and enhancing metabolic flexibility.

## 4.1 Conclusion

In conclusion, we observed elevated markers of mitophagy activity in skeletal muscle of ET compared with SED males, without a discernible difference in markers of autophagy. The consumption of a HFM was not associated with obvious changes in autophagy or mitophagy markers. Importantly, the greater content of mitophagy markers in skeletal muscle of ET individuals was not associated with a corresponding higher level of metabolic flexibility. However, skeletal muscle metabolic flexibility increased following the HFM in the ET but not SED individuals. Future studies are necessary to understand the role of skeletal muscle autophagy and mitophagy in metabolic health and disease, and in particular, to determine the mechanisms responsible for elevated mitophagy activity in skeletal muscle of ET individuals.

## Grants

This research was funded by ADA-07-12 (MWH).
